# X-box binding protein 1 (XBP1): a potential role in chemotherapy response, clinical pathologic features, non-inflamed tumour microenvironment for breast cancer

**DOI:** 10.1042/BSR20220225

**Published:** 2022-06-09

**Authors:** Zhipeng Zhu, Hongliang Zhan, Anran Sun, Heqing Huang, Baisheng Chen, Fuxing Zhang

**Affiliations:** 1 Department of General Surgery, The First Affiliated Hospital of Xiamen University, Xiamen, Xiamen Fujian, China; 2 Department of Gastrointestinal Surgery, The First Affiliated Hospital of Sun Yat-sen University, Guangzhou, Guangdong, China; 3 The School of Clinical Medicine, Fujian Medical University, Fuzhou Fujian, China; 4 Department of Urology, Foresea Life Insurance Guangzhou General Hospital, Guangzhou, Guangdong, China; 5 Endoscopy Center, Zhongshan Hospital of Fudan University (Xiamen Branch), Xiamen, Fujian, China

**Keywords:** Breast cancer, Molecular subtype, Neoadjuvant chemotherapy, Tumour microenvironment, XBP1

## Abstract

X-box binding protein 1 (XBP1) is mainly expressed in breast cancer (BC) in human cancers. Its tumorigenesis and favourable prognosis are contradictory, and its essential role in chemotherapeutic response and immunosuppression is unknown in BC. The study firstly identified XBP1 who received neoadjuvant chemotherapy (NAC) from GSE25055 and GSE24460. Associations between XBP1 expression and clinicopathological characteristics was investigated using Oncomine, TCGA, UALCAN and bc-GenExMiner. The prognostic value of XBP1 was assessed using the Kaplan–Meier Plotter, bc-GenExMiner, GSE25055, and GSE25056. Furthermore, we systematically correlated XBP1 and immunological characteristics in the BC tumour microenvironment (TME) using TISIDB, TIMER, GSE25055, GSE25056 and TCGA dataset. Finally, an essential role of XBP1 in chemotherapy response was evaluated based on GSE25055, GSE25065, GSE24460, GSE5846, ROC Plotter and CELL databases. Furthermore, XBP1 mRNA expression levels were obviously highest in BC among human cancers and were significantly related to a good prognosis. In addition, XBP1 mRNA and protein levels were higher in the luminal subtype than in normal tissues and basal-like subtype, which might be attributed to membrane transport-related processes. Apart from BC, negative immunological correlations of XBP1 were not observed in other malignancies. XBP1 might shape the non-inflamed TME in BC. Finally, XBP1 expression was higher in chemo-resistive than chemo-sensitive cases, it had a predictive value and could independently predict chemotherapy response in BC patients receiving NAC. Our study suggests that the essential role of XBP1 in clinical pathologic features, non-inflamed TME, chemotherapy response in BC.

## Introduction

Breast cancer (BC) remains the second most common cancer worldwide and is still the most common cancer among women [1]. According to statistical data, locally advanced BC accounted for approximately 54%, whose tumour is greater than 5 cm in size, possibly with the involvement of regional lymph nodes, chest walls, or skin [2]. Neoadjuvant chemotherapy is the standard treatment for locally advanced BC [3]. However, only 10–40% of BC patients demonstrate pathological complete response that is a prognostic indicator for long-term disease-free and overall survival [4,5]. Patients have a high risk of developing chemo-resistance during treatment through still unknown mechanisms [6]. Thus, it is critical to discover novel chemotherapy-resistant genes, which is required for developing new chemotherapy targets for BC.

Cell intrinsic and environmental fluctuations can dramatically influence the homeostasis of tumour cells, these stress conditions in the context of cancer cells can induce the accumulation of misfolded proteins. To cope with this condition, an adaptive mechanism to restore endoplasmic reticulum (ER) proteostasis is called the unfolded protein response (UPR) [7]. UPR is mainly mediated by three signalling pathways (IRE1a, PERK, and ATF6), IRE1a signalling is the most conserved pathway and function via mediating XBP1 to regulate diverse genes related to ER homeostasis. XBP1, a unique basic-region leucine zipper transcription factor involved in UPR that is important for cell survival to stress stimuli [8], is an emerging broad-spectrum target for cancer therapy. XBP1 is highly expressed in cells and tissues of various cancers and is widely involved in tumour progression and metastasis via regulating a diverse array of genes involved in cell survival, apoptosis, autophagy, metastasis, invasion, drug resistance, lipid metabolism and immunoregulation [9]. Lou et al. demonstrated that miR-199/ XBP1/cyclin D axis is important in the pathogenesis of hepatocellular carcinoma [10]. Chien et al. reported that targeting IRE1α/XBP1 might be a promising therapy [11]. In breast cancer, expression level of XBP1 influences the sensitivity of breast cancer to tamoxifen, and XBP1 increased sensitivity to tamoxifen in human breast cancer cell xenografts [12], and tumour autoimmune-related DCs with high expression of XBP1 can suppress antitumour immunity and promote the occurrence, invasion and metastasis of breast cancer [9]. However, it is critical to note that the potential role of XBP1 in efficacy of both standard chemotherapy and evolving cancer immunotherapies was not validated in BC [9], the role of XBP1 in BC should be further investigated.

In the study, we found that XBP1 is highest in BC among human cancers. We also reported that XBP1 could independently predict chemotherapy response in BC patients and promote the development of a non-inflamed TME in BC.

## Materials and methods

### Gene expression datasets

Gene expression profiles (GSE25055, GSE25065, GSE24460, and GSE5846) were obtained from the GEO database (http://www.ncbi.nlm.nih.gov/geo/). GSE25055 contained 113 NAC-resistant patients and 197 NAC-sensitive patients, GSE25056 contained 56 NAC-resistant patients and 142 NAC-sensitive patients, entire testing cohort contained 508 patients that were integrated from GSE25055 and GSE25065 using the MERGE function, random testing cohort contained 252 patients that were randomly selected from the entire testing cohort using the R package Caret with a ratio of 1:1 in a random manner, the detailed clinical information is shown in Table 1. Two cases of MCF-7 BC cell lines and two cases of the multistep doxorubicin-selected subline MCF-7/ADR were obtained from GSE24460. Docetaxel or paclitaxel-sensitive NCI-60 cell lines were obtained from GSE5846.

**Table 1:** Clinical characteristics of GSE25055, GSE25065, entire testing cohort and random testing cohort

Factor	GSE25055	GSE25065	Entire testing cohort	Random testing cohort
No. of patients	310	198	508	252
Age				
≤60	250	162	412	203
>60	60	36	96	49
ER status				
Negtive	135	75	210	113
Postive	175	123	298	139
PR status				
Negtive	167	97	264	139
Postive	143	101	244	113
Tumour size (cm)				
T0	2	0	2	1
T1	20	10	30	17
T2	165	90	255	123
T3	74	71	145	70
T4	49	26	75	41
Lymph node				
Negtive	87	70	157	83
Postive	223	128	351	169
Histological grade				
I	27	11	38	20
II	117	108	225	85
III	151	80	231	137
IV	15	0	15	10
NAC				
Sensitive	113	56	169	86
Resistant	197	142	339	166

Abbreviations: ER, Hormone receptors estrogen receptor; NAC, Neoadjuvant chemotherapy; No, Number; PR, Hormone receptors progesterone receptor.

Various cell lines from the Cancer Cell Line Encyclopaedia (CCLE) associated with chemoresistance and chemosensitivity were downloaded from CCLE (https://portals.broadinstitute.org/ccle/about), docetaxel-sensitive cell lines included LE: HL-60(TB), LE: RPMI-8226, BR: MDA-MB-435, CNS: SF-539, CO: COLO205, CO:HCC-2998, CO:HT29, and LC:NCI-H522; docetaxel-resistant cell lines included CNS: SF-268, RE: 786-0, RE: ACHN, RE:CAKI-1, OV: IGROV1, and OV: OVCAR-4; paclitaxel-sensitive cell lines included LE:RPMI-8226, BR: HS 578T, CNS: SF-539, CNS: SNB-75, CO: COLO 205, CO: HCC-2998, CO: HT29, and LC: NCI-H23; and paclitaxel-resistant cell lines included UN: NCI/ADR-RES; RE:A498; RE: UO-31; OV: OVCAR-4 ME: MALME-3M, ME: SK-MEL-28, and ME: UACC-257.

The raw RNA sequencing data, which comprises 1109 BC samples and 112 normal breast tissue samples, was selected from the TCGA dataset.

### Identification of NAC-related gene cluster

We used the R language to analyze the original CEL files of the GSE25055, GSE25065, GSE5846 and GSE24460 dataset.

The preprocessing procedures: Using the Bioconductor annotation package to convert microarray data probes into gene symbol, if multiple probes were mapped to a gene symbol, take the average value as the final expression value of the gene. Using the Limma R package to backgroundCorrect, Log2conversion, normalizeBetweenArrays, linear model desigh. Next, adjusted *P*<0.05 and |Log2 FC|>3 were used to select the differential gene expression (DEGs) between MCF7 BC and MCF-7/ADR cell lines from GSE24460 using “Limma” R package, adjusted *P*<0.05 and |Log2 FC|> 0.5 were used to select the DEGs between 113 NAC-resistant and 197 NAC-sensitive patients from GSE25055 using “Limma” R package.

Overlapped DEGs among GSE24460 and GSE25055 were identified using the OmicStudio tools(https://www.omicstudio.cn/tool). The PPI network of the overlapping DEGs was constructed using the Search Tool for the Retrieval of Interacting Genes (STRING; http://string.embl.de/), and a large gene cluster including six hub genes with the highest degree was extracted for further analysis. Among the six hub genes, the essential role of XBP1 in chemoresistance of BC is not well characterised and needs integrative analysis.

### Integrative analysis of XBP1

#### Expression analysis using a bioinformatics approach

The XBP1 mRNA expression profile was demonstrated in samples of 20 cancer types and matched non-tumour samples using Oncomine (https://www.oncomine.org) [13]. The mRNA expression levels of XBP1 in BC were validated using TIMER (https://cistrome.shinyapps.io/timer/) [14], GEPIA (http://gepia.cancer-pku.cn/) [15] and bc-GenExMiner (http://bcgenex.centregauducheau.fr/BC-GEM/) [16]. Moreover, the protein expression of XBP1 in BC was evaluated using UALCAN (http://ualcan.path.uab.edu/analysis.html) [17].

#### Survival analysis of XBP1 in BC

The effect of XBP1 expression level on survival outcome for all BC patients and different molecular subtypes was evaluated using TIMER [14], bc-GenExMiner databases, and Kaplan–Meier Plotter (http://kmplot.com/analysis/) [18]. Co-expression analysis was performed to obtain co-expression genes of XBP1 using LinkedOmics (http://www.linkedomics.org/) [19], In addition, Kaplan–Meier analysis, univariate and multivariate Cox proportional hazards analyses were performed to investigate the independence of XBP1 in BC based on GSE25055, GSE25065, random testing cohort, entire testing cohort and TIMER [14].

#### Essential role of XBP1 in tumour microenvironment

We determined the association between XBP1 and tumour immune components across human cancers using TISIDB7 [20]. We interactively explore the associations between immune infiltrates and XBP1 using 6 major analytic modules across 32 cancer types. Single-sample gene set enrichment analysis (ssGSEA) was performed to estimate a score for each case based on 29 immune-related gene sets and immune-related signatures. Genes in immune-related gene sets are shown in Supplementary Data S1. Stromal and immune scores of each case were calculated to verify the TME of different XBP1-defined groups using CIBERSORT computational method and the “ESTIMATE” package. Several immune-associated signatures [21] and immunotherapy-predicted pathways signature [22] were collected to assess immunological characteristics by ssGSEA.

#### Essential role of XBP1 in chemotherapy response

XBP1 expression profile was evaluated in cases of chemoresistive and chemosensitive cases from GSE25055, GSE25065, GSE24460, GSE5846, random testing cohort, entire testing cohort and CELL database (https://sites.broadinstitute.org/ccle). The chi-square test, univariate and multivariate logistic regression analyses and ROC Plotter (http://www.rocplot.org/) were performed to demonstrate the essential role of XBP1 in chemotherapy response. Based on BC patients treated NAC from GSE25055, Co-expression analysis was also performed to identify significant genes associated with XBP1 with *P*<0.01 and spearman correlation coefficient *r* > 0.4 or <-0.4, the positive and negative Co-expression genes were enriched using KOBAS (http://bioinfo.org/kobas/) and the OmicStudio tools (https://www.omicstudio.cn/tool). Gene set enrichment analysis (GSEA) was performed to identify the KEGG signalling pathways associated with XBP1.

#### Tissue microarray and immunohistochemistry (IHC) staining

A human breast cancer tissue microarray (TMA) was purchased from Shanghai OUTDO Biotech Co. Ltd (HBreD080CS01). All detailed clinical information including pathology, diagnosis, stage, ER level, PR level, HER-2 level and Ki-76 level is freely available on the Web (http://www.superchip.com.cn/biology/tissue.html). The anti-XBP1 antibody (Cat# 49436, signalway Antibody, U.S.A.) were used 1:100 dilution. TMA was scanned and imaged by Pannoramic DESK (3D HISTECH, HU), calculated by Quant Center2.1 (3D HISTECH, HU) software for positive cell ratio. The results were reviewed by two blinded pathologists.

### Statistical analysis

All statistical analyses were performed with R (V 3.6.3). and R package ggplot2 was used to visualize expression differences. The difference in the expression of XBP1 between two groups for our clinical samples was examined by Mann–Whitney test. Logistic regression analyses were done to evaluate the association between XBP1 expression and the clinical characteristics. The relationship between clinicopathologic variables and DRFS was analysed by Cox regression analyses. We drew the receiver operating characteristic (ROC) curve via the pROC package to evaluate the diagnostic efficacy of XBP1 expression. *P*-value < 0.05 was considered statistically significant.

## Results

### Identification of NAC-related gene cluster and XBP1

**Figure 1:**
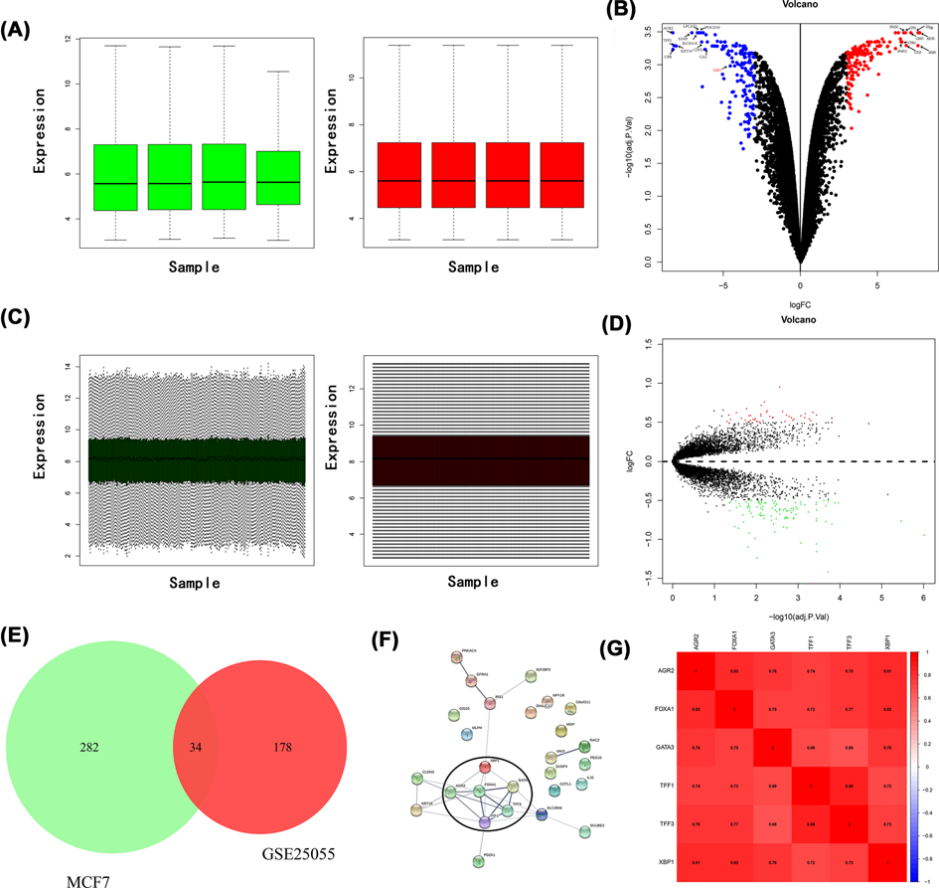
Identification of neoadjuvant chemotherapy related gene cluster and XBP1 (**A**) Normalization plot of expression profile form GSE24460, the green bar represents the data before normalization, and the red bar represents the normalized data. (**B**) Volcano plot of DEGs of GSE24460. The red points represent high expression genes, the blue points represent low expression genes, the black points represent genes with no significant difference (FDR<0.05, absolute log FC>3). (**C**) Normalization plot of expression profile form GSE25055, the green bar represents the data before normalization, and the red bar represents the normalized data. (**D**) Volcano plot of DEGs of GSE25055, the red points represent high expression genes, the green points represent low expression genes, the black points represent genes with no significant difference (FDR<0.05, absolute log FC>0.5). (**E**) Venn plot showing 34 genes shared in the intersection of GSE25055 and GSE24460. (**F**) The functional association network of overlapped genes was analyzed using the STRING database. (**G**) Heatmap demonstrated the correlation of genes in NAC-related gene cluster.

By performing DEGs analysis, 162 up-regulated and 154 down-regulated genes were obtained from GSE24460 (Figure 1A,B). In addition, 58 up-regulated and 154 down-regulated genes were identified from GSE25055 (Figure 1C,D). In total, 34 overlapping DEGs were identified between GSE25055 and GSE24460 datasets (Figure 1E). These genes were further used to construct PPI networks to obtain a large gene cluster including six hub genes with the highest degree (Figure 1F). Correlation heatmap revealed the six genes were highly correlated with each other (Figure 1G, all *r* > 0.7). Among the genes in the cluster, TFF3 [23,24], TFF1 [24,25], FOXA1 [26], GATA3 [27], and AGR2 [28,29] were reported to be associated with chemoresistance. However, the functional role of XBP1 in BC chemoresistance has not been well characterized and the potential value of XBP1 in BC should be further investigated.

**Figure 2:**
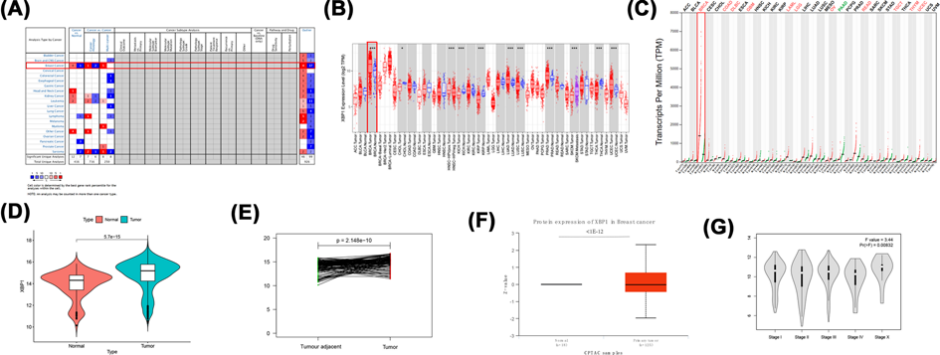
Overexpression of XBP1 in breast cancer (**A**) ONCOMINE; (**B**) TIMER; (**C**) GEPIA; (**D,E**) The mRNA expression of XBP1 was higher in breast cancer tissues than that in healthy tissues or adjacent breast tissues (TCGA); (**F**) The protien expression of XBP1 was higher in breast cancer tissues than that in healthy tissues (UALCAN); (**G**) Violin plot of XBP1 expression according to tumour stage (GEPIA). Represent unpaired *t*-test was performed.

### High XBP1 mRNA expression indicated good prognosis in BC patients

**Figure 3:**
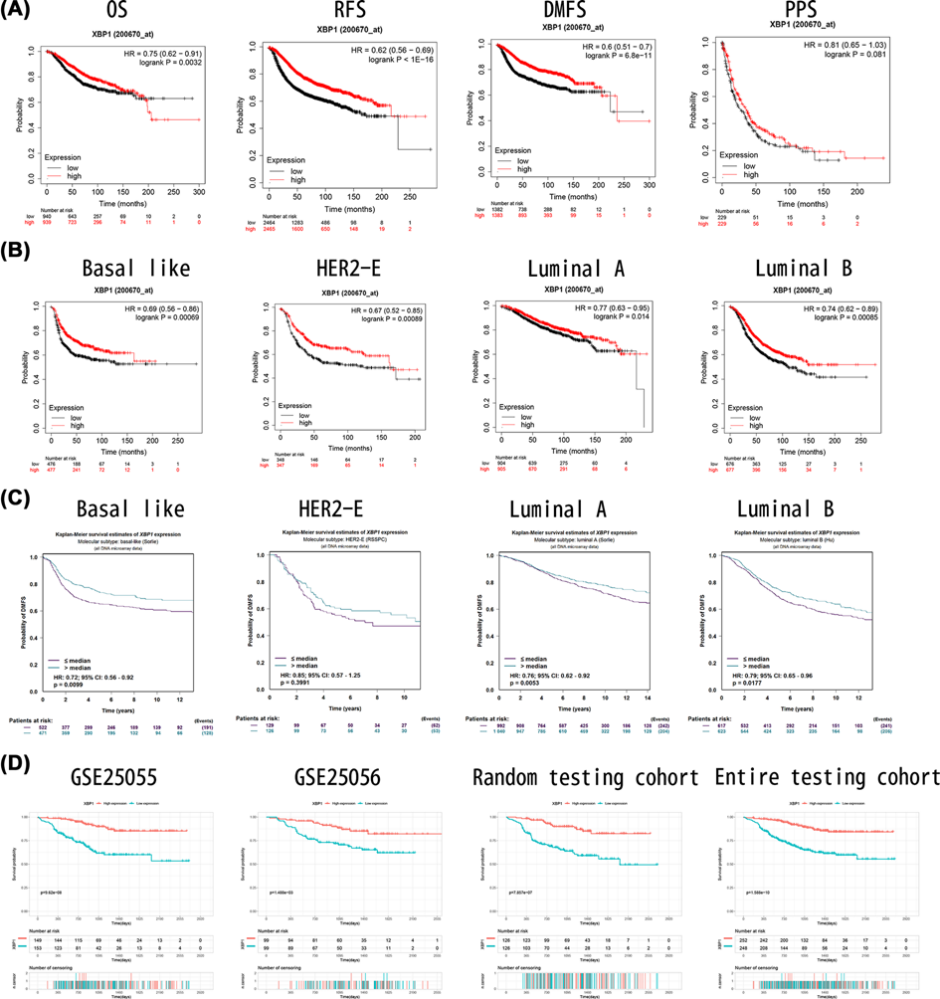
Prognostic value of XBP1 mRNA expression in patients with breast cancer (**A**) High XBP1 expression levels were significantly associated with better good prognosis in all BC patients based on Kaplan–Meier Plotter tool. (**B**) High XBP1 expression levels were significantly associated with better RFS in different molecular subtypes based on Kaplan–Meier Plotter tool. (**C**) High XBP1 expression levels were significantly associated with better DMFS in different molecular subtypes based on bc-GenExMiner. (**D**) High XBP1 expression levels were significantly associated with better DRFS in patients underwent neoadjuvant chemotherapy; BC, breast cancer; DMFS, distant metastasis-free survival; RFS, recurrence-free survival.

XBP1 mRNA expression levels were the highest in BC among human cancers (Figure 3A–C). XBP1 mRNA levels in tumour tissue were higher than in normal (Figure 3D) and tumour adjacent tissues (Figure 3E). In addition, the protein expression pattern of XBP1 was significantly different between BC primary and normal tissues (Figure 3F). XBP1 mRNA expression level was correlated with tumour stage (Figure 3G).

We assessed the association between XBP1 mRNA expression level and survival outcomes. High XBP1 expression levels were significantly associated with better overall survival (OS, Figure 3A), recurrence-free survival (RFS, Figure 3B), distant metastasis-free survival (DRFS, Figure 3C) and post-progression survival (PPS, Figure 3D) using the Kaplan–Meier Plotter tool. Meanwhile, high XBP1 expression levels were significantly associated with better RFS in basal-like patients (Figure 3E), HER2-E (Figure 3F), luminal A patients (Figure 3G) and luminal B patients (Figure 3H). We further performed bc-GenExMiner databases to explore the survival results in different molecular subtypes: high XBP1 expression levels were associated with better distant metastasis-free survival (DMFS) in basal-like patients (Figure 3I), HER2-E (Figure 3J), luminal A patients (Figure 3K) and luminal B patients (Figure 3L). Besides, high XBP1 expression was obviously associated with better distant recurrence-free survival (DRFS) (Figure 3M–P)

As shown in Table 2, univariate and multivariate cox regression analyses showed that only XBP1 was independently associated with DRFS. The Cox proportional hazard model also demonstrated that XBP1 was an independent favourable predictor of OS (Table 3).

**Table 2:** Univariate and Multivariate Cox-regression for DRFS in BC patients

Factors	Univariate Cox-regression			Multivariate Cox-regression		
	HR	95%CI	P-value	HR	95%CI	*P*-value
GSE25055						
Age	1.01	0.98–1.03	0.37			
ER status	0.33	0.20–0.55	2.58E-5	0.77	0.35–1.68	0.51
PR status	0.41	0.24–0.70	0.001	1.28	0.61–2.67	0.50
Tumour size (cm)	1.52	1.15–1.00	0.002	1.33	1.01–1.75	0.04
Lymph node	1.75	1.38–2.22	2.91E-6	1.56	1.21–2.02	0.00058
Histological grade	1.73	1.19–2.50	0.003	1.25	0.80–1.95	0.31
XBP1	0.25	0.14–0.44	1.63E-6	0.32	0.15–0.67	0.002
GSE25065–						
Age	0.97	0.94–1.005	0.108			
ER status	0.39	0.21–0.74	0.003	0.64	0.29–1.39	0.26
PR status	0.36	0.18–0.70	0.002	0.55	0.24–1.25	0.15
Tumour size(cm)	1.57	1.04–2.37	0.031	1.44	0.96–2.16	0.07
Lymph node	1.49	1.06–2.11	0.021	1.39	1.01–1.92	0.04
Histological grade	1.50	0.87–2.57	0.138			
XBP1	0.38	0.19–0.74	0.004	0.58	0.27–1.21	0.14
Random testing cohort						
Age	0.9894	0.9648–1.0148	0.410785			
ER status	0.3619	0.2144–0.6111	0.000143	0.8794	0.4465–1.7321	0.710144
PR status	0.2851	0.1568–0.5183	3.88E-05	0.6492	0.2868–1.4695	0.425007
Tumour size(cm)	1.5897	1.1905–2.1226	0.001676	1.4338	1.0771–1.9084	0.025841
Lymph node	1.5494	1.2052–1.9918	0.000635	1.2855	0.9916–1.6665	0.057905
Histological grade	1.7047	1.1138–2.5534	0.009670	1.2231	0.7759–1.9278	0.385742
XBP1	0.2555	0.1424–0.4581	4.64E-06	0.4151	0.1946–0.8855	0.022939
Entire testing cohort						
Age	0.99	0.97–1.01	0.61			
ER status	0.34	0.22–0.50	1.22E-07	0.68	0.39–1.18	0.17
PR status	0.36	0.23–0.55	3.57E-06	0.92	0.53–1.59	0.78
Tumour size (cm)	1.65	1.30–2.08	2.43E-05	1.38	1.10–1.73	0.004
Lymph node	1.62	1.33–1.97	1.14E-06	1.48	1.22–1.81	0.00007
Histological grade	1.69	1.24–2.31	0.0009	1.07	0.76–1.50	0.68
XBP1	0.29	0.19–0.44	1.12E-08	0.39	0.23–0.66	0.0005

Abbreviations: BC, breast cancer; DRFS, distant recurrence-free survival; ER, hormone receptors estrogen receptor; PR, hormone receptors progesterone receptor; XBP1, X-box binding protein 1.

**Table 3:** The Cox proportional hazard model of XBP1 and clinical factors in BC (TIMER)

Factor	coef	HR	95%CI_l	95%CI_H	P_value	Sig
Age	0.037	1.038	1.022	1.053	0.000	***
Gender_male	0.276	1.318	0.181	9.605	0.785	
Race_Black	-0.4	0.671	0.196	2.3	0.525	
Race_White	-0.583	0.558	0.172	1.808	0.331	
Stage2	0.43	1.538	0.806	2.933	0.191	
Stage3	1.306	3.69	1.901	7.16	0.000	***
Stage4	2.623 1	3.772	6.094	31.126	0.000	***
Purity	0.348	1.417	0.493	4.073	0.518	
B_cell	-0.693	0.5	0.005	54.039	0.772	
CD8_Tcell	-1.39	0.249	0.018	3.416	0.298	
CD4_Tcell	0.508	1.662	0.034	82.159	0.799	
Macrophage	3.196 2	4.43	1.448	412.043	0.027	*
Neutrophil	2.163	8.695	0.034	2239.329	0.445	
Dendritic	-0.913	0.401	0.048	3.382	0.401	
XBP1	-0.199	0.819	0.725	0.926	0.001	**

Abbreviations: BC, breast cancer; XBP1, X-box binding protein 1; * to indicate *P*<0.05; ** to indicate *P*<0.01; *** to indicate *P*<0.001.

### Co-expression and functional enrichment analysis based on BC patients

**Figure 4:**
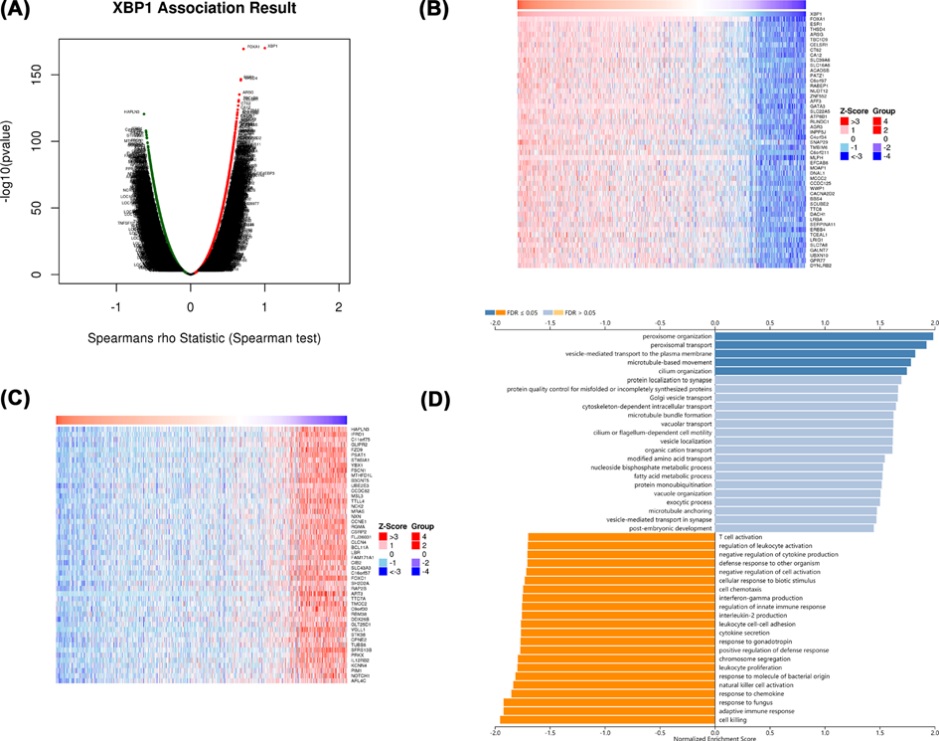
Co-expression analysis and gene set enrichment analysis in breast cancer using Linkedomics database (**A**) Volcano plot showed positive and negative related genes of XBP1 in breast cancer. (**B,C**) Heatmap of the top 50 positively and negatively correlated genes of XBP1 in breast cancer. (**D**) Gene set enrichment analysis for all positively and negatively correlated significant genes of XBP1.

Co-expression analysis was performed to understand the underlying roles of XBP1 in BC. The volcano plot showed genes correlated with XBP1 (Figure 4A). The heatmap presented the top 50 positive and negative XBP1 genes (Figure 4B,C). GSEA was performed based on all significantly related genes (Figure 4D). XBP1 was positively associated with membrane transport and formulation-related processes, including peroxisome organisation, peroxisomal transport, microtubule-based movement. XBP1 was negatively associated with immunity-related biological processes, including adaptive immune response, response to chemokines and T-cell activation.

### Association between XBP1 expression and clinicopathological characteristic in BC patients

**Figure 5:**
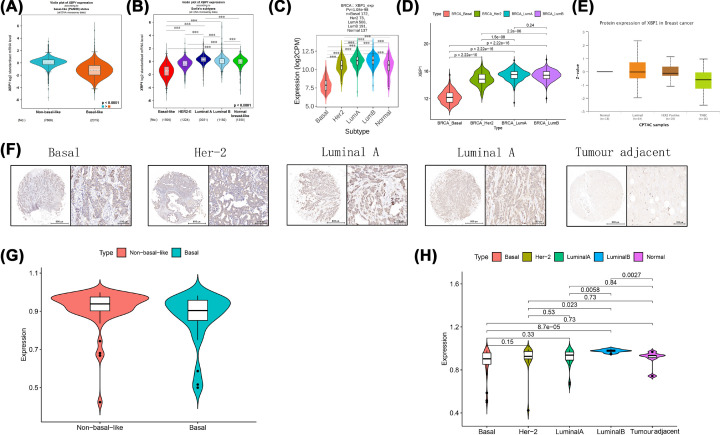
The expression of XBP1 in breast cancer with different molecular subtypes in different datasets (**A,B**) bc-GenExMiner; (**C**) TISIDB7; (**D**) TCGA; (**E**) UALCAN; (**F**) Representative images show XBP1 protein expression in different molecular subtypes using tissue microarray slides; (**G,H**) Tissue microarray slides.

XBP1 was significantly higher in the non-basal-like group than in the basal-like group (Figure 5A), and XBP1 expression was obviously overexpressed in luminal BC using the (Figure 5B–D). Meanwhile, the protein expression level of XBP1 was overexpressed in the luminal type and downregulated in basal-like tissues (Figure 5E). For further validation, we also detected the expression of XBP1 in clinical BC samples, the XBP1 expression levels were significantly higher in luminal BC tissues and lower in basal-like BC tissues than that in tumour adjacent tissues (Figures 5F and 7H). Moreover, compared with non-basal-like tissues, the XBP1 expression was obviously down-regulated in basal-like tissues (Figure 5G). GSEA indicated that XBP1 was positively correlated with membrane transport and formulation, which provided a biological mechanism for clinicopathological characteristics.

### High XBP1 mRNA expression indicated non-inflamed tumour microenvironment of BC patients

The co-expressed and functional enrichment analysis showed that XBP1 was correlated with immune exhaustion in BC. We further investigated the role of XBP1 in immune characteristics, XBP1 was negatively correlated with the infiltration level of tumour-infiltrating immune cells (TILs) in BC across human cancers (Supplementary Figure S1A). Furthermore, XBP1 expression was negatively correlated with almost all immunological biomarkers and TILs in BC patients across human cancers (Supplementary Figure S1B). The profile indicated that XBP1 participated in the immune suppression process and played a vital role in the immuno-oncological interactions of BC.

**Figure 6:**
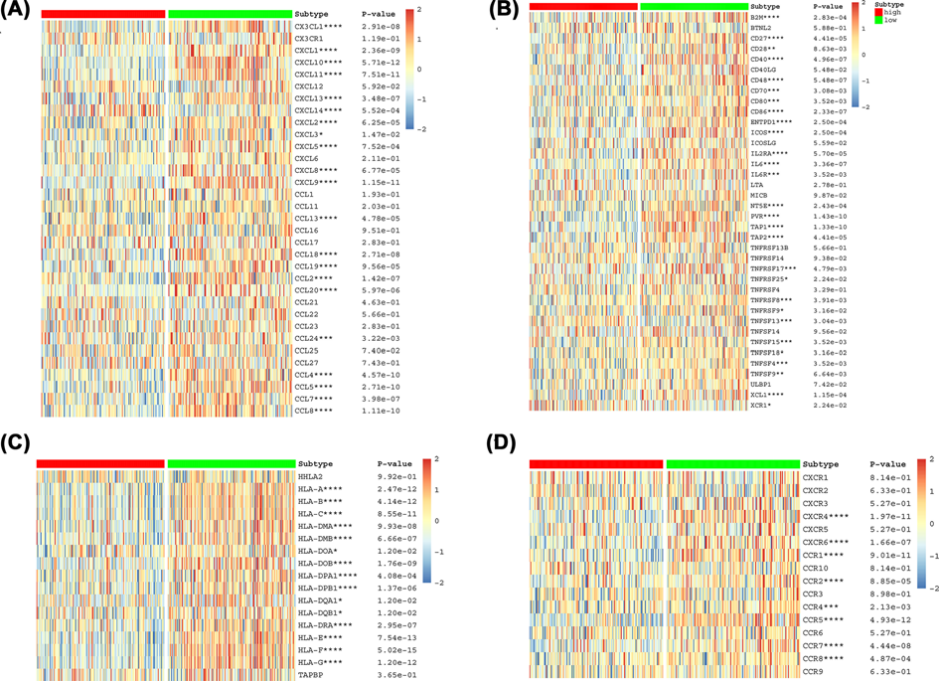
Correlation between XBP1 and immunomodulators based on GSE25055 (**A**) chemokines; (**B**) immunostimulators; (**C**) MHC; (**D**) Receptors.

**Figure 7:**
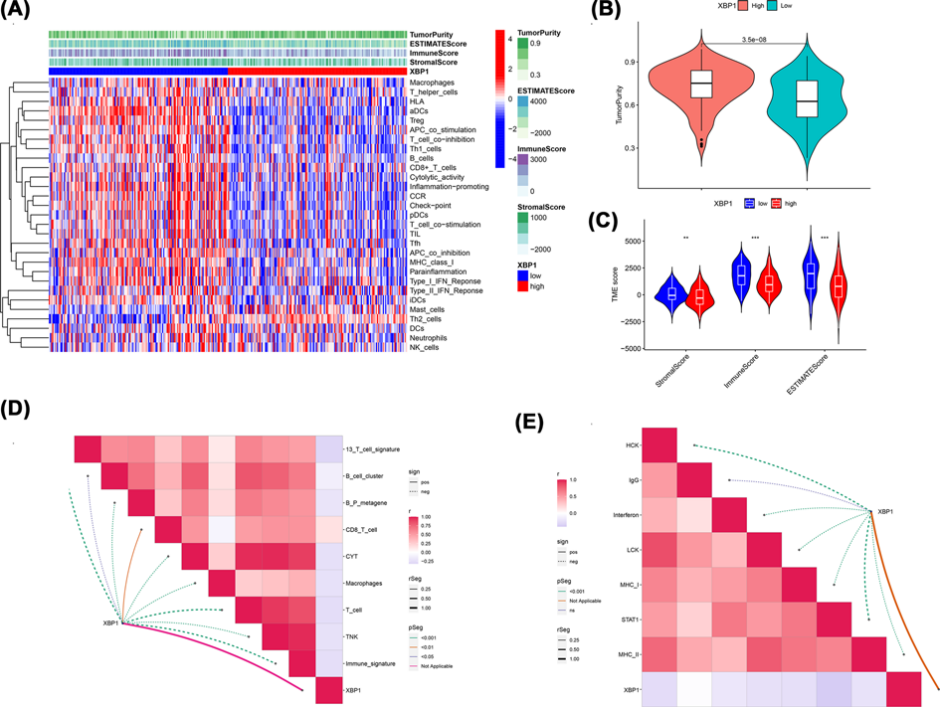
XBP1 shapes a non-inflamed TME in BC based on GSE25055 (**A**) Scores of the 29 immune-related gene sets between high and low XBP1 group. (**B**) Difference in TumorPurity between high and low XBP1 group. (**C**) Difference in TME score between high and low XBP1 group. (**D**) Correlations between XBP1 and the enrichment scores of immunocytes. (**E**) Correlations between XBP1 and the enrichment scores of inflammatory activation functions.

XBP1 was negatively associated with various immunological biomarkers (Figure 6A). Most MHC molecules were down-regulated in the high-XBP1 group, which indicated a down-regulated antigen presentation function (Figure 6C). Most chemokines and receptors, which could function to recruit CD8+ T cells, TIIC and Th17 into TME in BC, were down-regulated in XBP1 high group (Figure 6B,D). Compared with low-XBP1 group, BC patients in the high-XBP1 group had lower levels of immune-related gene sets (Figure 7A), lower tumour purity (Figure 7B) and higher immune scores and higher stromal scores (Figure 7C).

We hypothesized that XBP1 could shape a non-inflamed TME for BC. The enrichment score of inflammatory response-related signatures was calculated to assess the inflamed status [30]. XBP1 was negatively associated with enrichment of immunocytes, including T cells (13 T cell signature, T cells, CD8+ T cells and T cells. Metagene), B cells (B cell clusters and B.P. metagene), macrophages and cytolytic activity score (CYT) (Figure 7D). XBP1 was also negatively associated with HCK, LCK, MHC-I and IgG, indicating that XBP1 inhibited inflammatory activation functions in BC, including suppression of macrophage, B, and T cell signaling transduction (Figure 7E).

**Figure 8:**
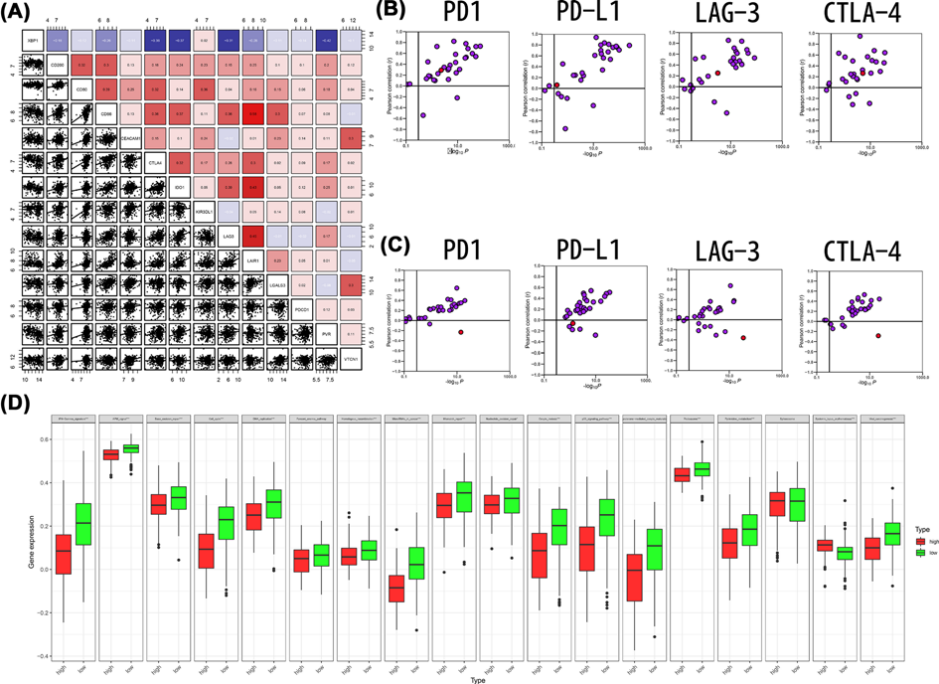
Correlation between XBP1 and inhibitory immune checkpoints (**A**) Correlation between XBP1 and 13 inhibitory immune checkpoints. (**B**) Correlation between XBP1 and four immune checkpoints (PD-L1, CTLA-4, PD-1 and LAG-3) based on GETx dataset. (**C**) Correlation between XBP1 and four immune checkpoints (PD-L1, CTLA-4, PD-1 and LAG-3) based on TCGA dataset. The dots represent cancer types or tissue types, red dots represent BC or breast tissue. The *Y*-axis represents the Pearson correlation, while the *X*-axis represents -log_10_*P*. (**D**) Differences in the enrichment scores of immunotherapy-predicted pathways between high and low XBP1 groups.

The up-regulation of inhibitory immune checkpoints is a critical characteristic of an inflamed TME, which suppresses excessive immune responses. XBP1 was mutually exclusive of a major inhibitory immune checkpoint (Figure 8A). XBP1 was positively associated with immune checkpoint inhibitors (PD-L1, PD-1, CTLA-4 and LAG-3) in normal breast tissue based on the GTEx dataset (Figure 8B). However, XBP1 was negatively correlated with immune checkpoint inhibitors in BC based on the TCGA dataset (Figure 8C), and these negative immunological correlations of XBP1 were not observed in other malignancies. The down-regulated expression of inhibitory immune checkpoints might be attributed to the non-inflamed TME shaped by XBP1 in BC. In addition, XBP1 was negatively correlated with the enrichment scores of most immunotherapy-positive gene signatures (Figure 8D), which further proved the essential role of XBP1 in non-inflamed TME formation.

These role of XBP1 to shape non-inflamed tumour microenvironment in BC was validated in GSE25065 (Supplementary Figures 2,4,5) and TCGA dataset (Supplementary Figures 3,4,5).

### High XBP1 expression indicated high chemosensitivity

**Figure 9:**
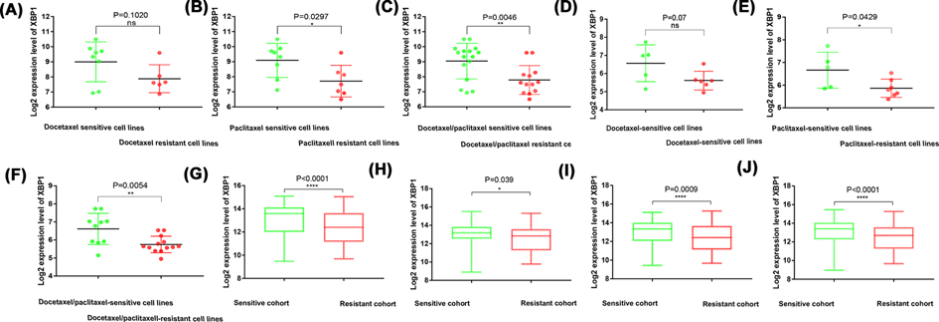
High XBP1 Mrna expression level in chemo-resistive cases (**A–C**) NCI-60 cell lines; (**D–F**) CCLE; (**G**) GSE25055; (**H**) GSE25065; (**I**) Random testing cohort; (**J**) Entire testing cohort.

XBP1 mRNA expression levels were higher in docetaxel-sensitive cell lines than in docetaxel-resistant cell lines from NCI-60 cell lines (Figure 9A) and CCLE (Figure 9D). XBP1 mRNA expression levels were significantly higher in paclitaxel-resistant cell lines than in paclitaxel-sensitive cell lines from NCI-60 cell lines (Figure 9B) and CCLE (Figure 9E). In addition, XBP1 mRNA expression levels were also significantly higher in taxane-sensitive cell lines than in taxane-resistant cell lines from NCI-60 cell lines (Figure 9C) and CCLE (Figure 9F). Furthermore, we found that XBP1 expression levels were significantly higher in the NAC-sensitive cohort than in the NAC-resistant cohort (Figure 9G–J)

To furtherly explore the clinically predictive value of NAC response of XBP1, ROC analysis was performed to assess the clinically predictive value of NAC response among XBP1 and other clinical characteristics (age, ER status, PR status, tumour size (cm), lymph node and histological grade), and the AUC value of XBP1 for NAC response was obviously greater than other clinical characteristics (Table 4). As determined using the ROC Plotter tool based on 2108 BC patients treated with chemotherapy, AUC of chemotherapy response of XBP1 is 0.569 with *P*-value < 4.10E-05, which is similar to the genes which are known as chemotherapy resistance-related genes (Table 5), demonstrated predictive value of published biomarkers for chemotherapy response in BC using ROC Plotter [6,31–52]. Therefore, it revealed that XBP1 has predictive value for chemotherapy response in BC treated with chemotherapy.

**Table 4:** The ROC analysis indicated AUC value of XBP1 for NAC response was higher than other clinical characteristics

	GSE25055	GSE25065	Random testing cohort	Entire testing cohort
XBP1	0.669	0.615	0.643	0.624
Age	0.519	0.528	0.516	0.521
ER status	0.598	0.53	0.531	0.571
PR status	0.579	0.558	0.557	0.569
Tumour size (cm)	0.536	0.524	0.528	0.534
Lymph node	0.593	0.57	0.599	0.599
Histological grade	0.56	0.549	0.516	0.557

Abbreviations: ER, hormone receptors estrogen receptor; NAC, neoadjuvant chemotherapy; PR, hormone receptors progesterone receptor; XBP1, X-box binding protein 1.

**Table 5:** ROC analysis demonstrated predictive value of published biomarkers for chemotherapy response in BC using ROC Plotter

Author	Biomarker	ROC *P*-value	TPR	RNR	AUC
	XBP1	4.10E-05	0.5	0.62	0.569
Diana E Baxter [42]	ABCG2	0.00000016	0.52	0.6	0.576
Yu Wang [51]	PKM2	0.000041	0.64	0.51	0.572
Mohammad Sultan [40]	BCL6	0.00087	0.53	0.57	0.549
Shanshan Sun [49]	PGK1	0.27	0.56	0.48	0.509
Yuanyuan Cheng [31]	ADAM10	2.2E-11	0.6	0.56	0.601
Xiyu Liu [50]	SYTL4	0.065	0.45	0.67	0.545
Mariko Nishie [35]	ATP6V1B1	0.003	0.5	0.58	0.541
Hengxing Chen [43]	PARK2	0.000082	0.58	0.54	0.556
Sandra Zazo [33]	CCL5	0.00000033	0.54	0.61	0.589
Qingjian Li [47]	rac1	0.0028	0.63	0.55	0.589
Waleed S Al Amri [37]	MUC17	0.007	0.61	0.57	0.581
Yuhong Li [52]	ASAH1	0.00004	0.42	0.7	0.557
Ryuji Ohashi [45]	IMP3	0.069	0.58	0.49	0.522
Pinto JA [39]	DDIT4	0.0000016	0.59	0.53	0.569
Sujin Yang [6]	GSTP1	0.0000019	0.54	0.61	0.57
Mamoru Takada [34]	BRCA1	0.00018	0.5	0.58	0.555
TMA Abdel Fatah [38]	DDX43	0.37	0.46	0.55	0.505
	SERPINA6	1.7E-09	0.58	0.54	0.588
	BEX1	3.7E-12	0.56	0.59	0.6
	SLC26A3	2.7E-13	0.58	0.57	0.608
	LAPTM4B	0.00086	0.67	0.44	0.546
Justin M Balko [44]	DUSP4	0.14	0.45	0.58	0.516
Carole Massabeau [36]	FGFR1	0.0093	0.55	0.51	0.535
	FKBP4	0.0021	0.53	0.54	0.544
Won Suk Yang [46]	S100A9	0.066	0.48	0.57	0.523
Gottfried E Konecny [32]	TOP2A	0.0011	0.56	0.53	0.547
Heidi Fiegl [41]	NEUROD1	7E-13	0.53	0.62	0.603

Abbreviations: AUC, Area under curve; BC, breast cancer; RNR, true negtive rate; ROC, receiver operating characteristic; TPR, true positive rate.

We investigated whether the expression level of XBP1 has guiding significance for clinicopathological work by using logistic regression to analyze the predictive value of XBP1. Univariate logistic-regression showed that high XBP1 mRNA expression level was associated with good response. Multivariate logistic regression revealed that XBP1 expression was independently associated with chemotherapy response in BC patients (Table 6).

**Table 6:** Univariate and Multivariate logistic-regression for response to NAC in BC patients

Factors	Univariate logistic-regression`			Multivariate logistic-regression		
	OR	95%CI	*P*-value	OR	95%CI	*P*-value
GSE25055						
Age	1.01	0.98–1.03	0.365	–	–	–
ER status	0.441	0.28–0.72	0.001	0.90	0.40-2.04	0.808
PR status	0.526	0.32–0.84	0.008	1.04	0.54-2.00	0.893
Tumour size (cm)	1.191	0.90–1.57	0.219		–	–
Lymph node	1.642	1.23–2.19	0.001	1.51	1.12-2.03	0.006
Histological grade	1.440	1.02–2.03	0.039	1.06	0.703-1.59	0.781
XBP1 (low vs high)	0.684	0.57–0.82	0.000045	0.73	0.56-0.95	0.019
GSE25065						
Age	1.013	0.982–1.044	0.414	–	–	–
ER status	1.516	0.923–2.491	0.100	–	–	–
PR status	1.413	0.933–2.140	0.103	–	–	–
Tumour size (cm)	1.184	0.781–1.796	0.426	–	–	–
Lymph node	1.413	0.933–2.14	0.103	–	–	–
Histological grade	1.516	0.92–2.49	0.100	–	–	–
XBP1 (low vs high)	0.430	0.22–0.83	0.012	0.430	0.22–0.83	0.012
Random testing cohort						
Age	1.006	0.98–1.03	0.653			
ER status	0.77	0.46–1.31	0.342			
PR status	0.63	0.37–1.07	0.086			
Tumour size (cm)	1.14	1.84–1.55	0.40			
Lymph node	1.52	1.10–2.08	0.01	1.40	1.01-1.96	0.042
Histological grade	1.18	0.82–1.72	0.37			
XBP1 (low vs high)	0.33	0.19–0.58	0.000089	0.36	0.20–0.63	0.000336
Entire testing group						
Age	1.01	0.99–1.03	0.178			
ER status	0.55	0.37–0.81	0.002	1.19	0.66–2.15	0.55
PR status	0.56	0.39–0.82	0.002	0.99	0.59–1.67	0.99
Tumour size (cm)	1.19	0.95–1.50	0.11			
Lymph node	1.53	1.21–1.93	0.0003	1.47	1.16–1.87	0.001
Histological grade	1.46	1.09–1.94	0.009	1.22	0.89–1.68	0.208
XBP1(low vs high)	0.38	0.25–0.55	8.45E-07	0.38	0.23–0.63	0.0002

Abbreviations: ER, hormone receptors estrogen receptor; NAC, neoadjuvant chemotherapy; PR, hormone receptors progesterone receptor; XBP1, X-box binding protein 1; Patients were divided into the high and low subgroups using the median XBP1 expression.

To better understand the clinical significance of XBP1 expression, we investigated the correlations between the expression level of XBP1 and BC clinicopathological parameters and found that the expression level of XBP1 was significantly correlated with NAC sensitivity (Table 7).

**Table 7:** Correlation of *XBP1* expression with clinicopathological characteristics based on GSE25055

Characteristic	XBP1		*P*-value
	Low	High	
No. of patients	155	155	
Age (years)			0.329
<60	126	119	
≥60	29	36	
ER status			3.8893E-31
Negative	118	17	
Positive	37	139	
PR status			3.6812E-23
Negative	127	40	
Positive	28	115	
Tumour size (cm)			0.458
I	11	9	
II	75	90	
III	41	33	
IV	26	23	
Lymph node			0.008
Negative	33	54	
Positive	122	101	
Histological grade			3.1306E-11
I	7	12	
II	31	86	
III	104	47	
IV	11	4	
NAC sensitivity			0.000013
Sensitive	38	75	
Resistant	117	80	

Abbreviations: ER, hormone receptors estrogen receptor; NAC, neoadjuvant chemotherapy; No, number; PR, hormone receptors progesterone receptor; XBP1, X-box binding protein 1; Patients were divided into the high and low subgroups using the median XBP1 expression.

### Co-expression and functional enrichment analysis based on BC patients treated using NAC

We identified co-expressed genes of XBP1 using expression data from GSE25055, 519 positively co-expressed genes (*R* > 0.4, *P* < 0.01) and 458 negatively co-expressed genes (*R* < -0.4, *P*<0.01) were obtained (Supplementary Data S2). XBP1 was positively associated with metabolic pathways, such as peroxisome, fatty acid metabolism, valine, leucine and isoleucine degradation (Supplementary Figure 6A,B) and negatively associated with the cell cycle, including DNA replication, urine metabolism, and RNA polymerase (Supplement Figure 6C,D). GSEA revealed that KEGG_CELL_CYCLE and KEGG_P53_SIGNALING_PATHWAY were enriched with low XBP1 expression, whereas KEGG-DRUG_METABOLISM_CYTOCHROME_P450 was enriched with high XBP1 expression (Supplement Figure 6E).

## Discussion

In the present study, XBP1 expression was the highest in BC across human cancers, and the mRNA and protein levels of XBP1 in BC were higher than that in normal tissue. Interestingly, mRNA and protein expression levels of XBP1 increased in the luminal subtype and decreased in the basal-like subtype. A previous study indicated that XBP1 is directly related to the ER signalling pathway and is involved in related genes [53–55], which can explain the high XBP1 expression in the luminal subtype. Meanwhile, GSEA showed that XBP1 was positively associated with membrane transport and formulation-related processes, which can also explain the increase in XBP1 expression in luminal subtypes.

We further investigated the immunological characteristics of XBP1, XBP1 expression was negatively correlated with almost all immunological biomarkers and TILs in BC patients across human cancers, and the result was further validated using data from GSE25055, GSE25056 and TCGA dataset, which also indicated that XBP1 was negatively associated with immune-related processes, such as adaptive immune response, response to chemokines, and T cell activation, suggesting that XBP1 might have an immunosuppressive role in BC. XBP1 was reported to exert inhibitory effects on protective T cell-mediated anti-cancer immunity [56–58]. However, our study showed that XBP1 could comprehensively down-regulate the expression of critical immunomodulators. Subsequently, the inflammatory response is down-regulated. Furthermore, the recruitment of effector TIICs decreased and shaped a non-inflamed TME for BC. Finally, the expression of the inhibitory immune checkpoints was downregulated. Therefore, XBP1 could shape a non-inflamed TME for BC.

XBP1 is upregulated in many types of cancers and correlated with a poor prognosis [59,60]. However, in the present study, high expression was correlated with good prognosis in BC patients receiving NAC and was validated using various databases, which is contradictory to the tumorigenic role of XBP1. There may be two reasons for the contradictory conclusions. First, the function of XBP1 is complicated for cell functions and can regulate multiple biological processes and signalling networks via 162 genes [61], and the various pathways in which XBP1 is involved is still obscure [61,62]. Secondly, our result showed that XBP1 expression was higher in chemotherapy-sensitive cases and that XBP1 was an independent predictive factor for NAC response in BC patients treated with NAC, suggesting XBP1 may regulate chemotherapeutic response to inhibit tumours. Routine chemotherapeutic mechanisms are known as microtubule stabilizers that decrease the frequency of detachment by arresting the cell cycle [62]. Co-expression analysis based on BC patients treated with NAC revealed that negatively correlated genes of XBP1 were mainly enriched in the cell cycle process. In addition, GSEA based on BC patients treated with NAC showed that high XBP1 expression was negatively related to the cell cycle process, indicating that XBP1 might act as a cell cycle process suppressor. Therefore, XBP1 might play a double-face role in the development and progression of cancers, and the molecular mechanism regulated by XBP1 is still obscure; Therefore, the different pathways that XBP1 is involved in need to be further investigated.

In summary, XBP1 expression level obviously increased in BC patients, especially in patients with luminal BC, and high XBP1 expression indicated high chemosensitivity, good prognosis, and a non-inflamed tumour microenvironment in BC patients. However, the molecular network regulated by XBP1 is still obscure and requires further laboratory research support.

## Supplementary Material

Supplementary Figures S1-S6

Supplementary Data S1-S2

## Data Availability

The original contributions presented in the study are publicly available. These data can be found here: GEO dataset (https://www.ncbi.nlm.nih.gov/gds and TCGA dataset (https://portal.gdc.cancer.gov/repository).

## Abbreviations

BC: breast cancer
CYT: cytolytic activity score
DEG: differential gene expression
DMFS: distant metastasis-free survival
DRFS: distant metastasis-free survival
ER: endoplasmic reticulum
GSEA: gene set enrichment analysis
NAC: neoadjuvant chemotherapy
OS: overall survival
PPS: post-progression survival
RFS: recurrence-free survival
ROC: receiver operating characteristic
ssGSEA: single-sample gene set enrichment analysis
TIL: tumour-infiltrating immune cell
TME: tumour microenvironment
TPR: true positive rate
UPR: unfolded protein response
XBP1: X-box binding protein 1
