# Supplementary material for: X-box binding protein 1 (XBP1): a potential role in chemotherapy response, clinical pathologic features, non-inflamed tumour microenvironment for breast cancer
Source: Biosci Rep. 2022 Jun 9;42(6):BSR20220225. doi: 10.1042/BSR20220225 (PMC9202509; doi:10.1042/BSR20220225)
Supplement: Supplementary Figures S1-S6 [file BSR-2022-0225_supp.pdf]

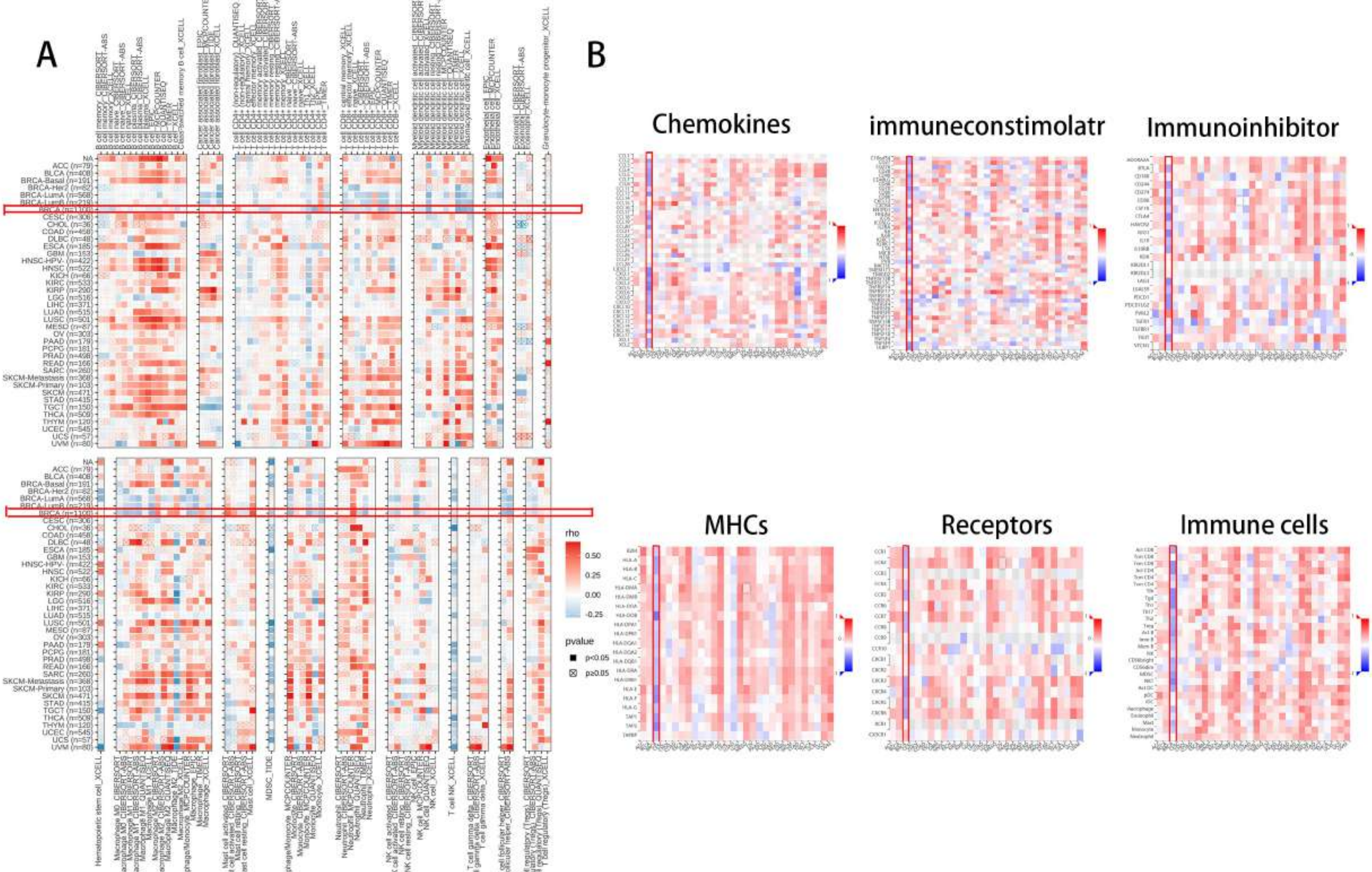
A
B
Chemokines
immuneconstimolatr
Immunoinhibitor
MHCs
Receptors
Immune cells
rho
0.50
0.25
0.00
-0.25
pvalue
p<0.05
p≥0.05

Supplement Figure 1: The effect of XBP1 on immunological status in pan-cancers. (A) The correlations of XBP1 expression and immune infiltration in pan-cancers using seven independent algorithms; (B) Correlations of XBP1 expression with immunosuppressive features of pan-cancers using TISIDB.

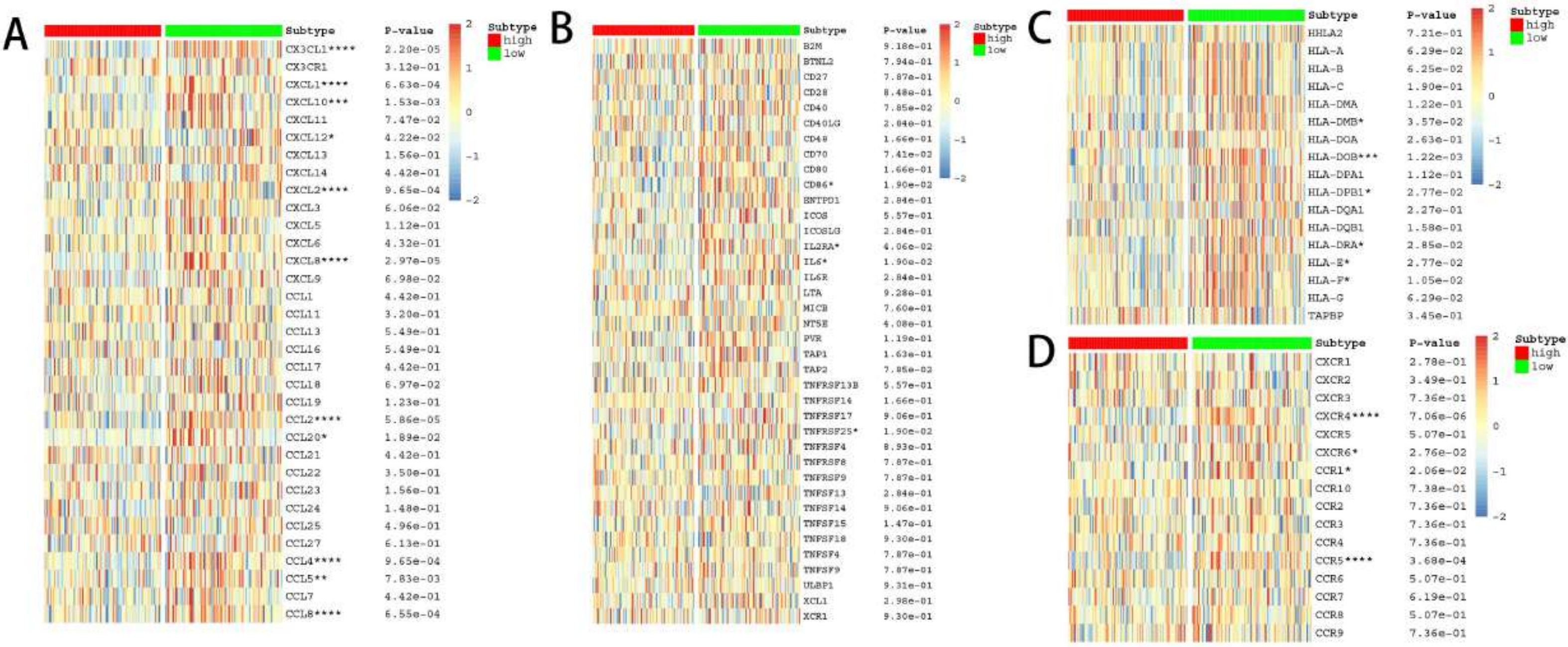
A
Subtype
P-value
CX3CL1**** 2.20e-05
CX3CR1 3.12e-01
CXCL1**** 6.63e-04
CXCL10*** 1.53e-03
CXCL11 7.47e-02
CXCL12* 4.22e-02
CXCL13 1.56e-01
CXCL14 4.42e-01
CXCL2**** 9.65e-04
CXCL3 6.06e-02
CXCL5 1.12e-01
CXCL6 4.32e-01
CXCL8**** 2.97e-05
CXCL9 6.98e-02
CCL1 4.42e-01
CCL11 3.20e-01
CCL13 5.49e-01
CCL16 5.49e-01
CCL17 4.42e-01
CCL18 6.97e-02
CCL19 1.23e-01
CCL2**** 5.86e-05
CCL20* 1.89e-02
CCL21 4.42e-01
CCL22 3.50e-01
CCL23 1.56e-01
CCL24 1.48e-01
CCL25 4.96e-01
CCL27 6.13e-01
CCL4**** 9.65e-04
CCL5** 7.83e-03
CCL7 4.42e-01
CCL8**** 6.55e-04
Subtype
high
low
2
1
0
-1
-2
B
Subtype
P-value
B2M 9.18e-01
BTNL2 7.94e-01
CD27 7.87e-01
CD28 8.48e-01
CD40 7.85e-02
CD40LG 2.84e-01
CD48 1.66e-01
CD70 7.41e-02
CD80 1.66e-01
CD86* 1.90e-02
ENTPD1 2.84e-01
ICOS 5.57e-01
ICOSLG 2.84e-01
IL2RA* 4.06e-02
IL6* 1.90e-02
IL6R 2.84e-01
LTA 9.28e-01
MICB 7.60e-01
NT5E 4.08e-01
PVR 1.19e-01
TAP1 1.63e-01
TAP2 7.85e-02
TNFRSF13B 5.57e-01
TNFRSF14 1.66e-01
TNFRSF17 9.06e-01
TNFRSF25* 1.90e-02
TNFRSF4 8.93e-01
TNFRSF8 7.87e-01
TNFRSF9 7.87e-01
TNFSF13 2.84e-01
TNFSF14 9.06e-01
TNFSF15 1.47e-01
TNFSF18 9.30e-01
TNFSF4 7.87e-01
TNFSF9 7.87e-01
ULBP1 9.31e-01
XCL1 2.98e-01
XCR1 9.30e-01
Subtype
high
low
2
1
0
-1
-2
C
Subtype
P-value
HHLA2 7.21e-01
HLA-A 6.29e-02
HLA-B 6.25e-02
HLA-C 1.90e-01
HLA-DMA 1.22e-01
HLA-DMB* 3.57e-02
HLA-DOA 2.63e-01
HLA-DOB*** 1.22e-03
HLA-DPA1 1.12e-01
HLA-DPB1* 2.77e-02
HLA-DQA1 2.27e-01
HLA-DQB1 1.58e-01
HLA-DRA* 2.85e-02
HLA-E* 2.77e-02
HLA-F* 1.05e-02
HLA-G 6.29e-02
TAPBP 3.45e-01
Subtype
high
low
2
1
0
-1
-2
D
Subtype
P-value
CXCR1 2.78e-01
CXCR2 3.49e-01
CXCR3 7.36e-01
CXCR4**** 7.06e-06
CXCR5 5.07e-01
CXCR6* 2.76e-02
CCR1* 2.06e-02
CCR10 7.38e-01
CCR2 7.36e-01
CCR3 7.36e-01
CCR4 7.36e-01
CCR5**** 3.68e-04
CCR6 5.07e-01
CCR7 6.19e-01
CCR8 5.07e-01
CCR9 7.36e-01
Subtype
high
low
2
1
0
-1
-2

Supplement Figure 2: Correlation between XBP1 and immunomodulators based on GSE25065. (A) chemokines; (B) immunostimulators; (C) MHC; (D) receptors.

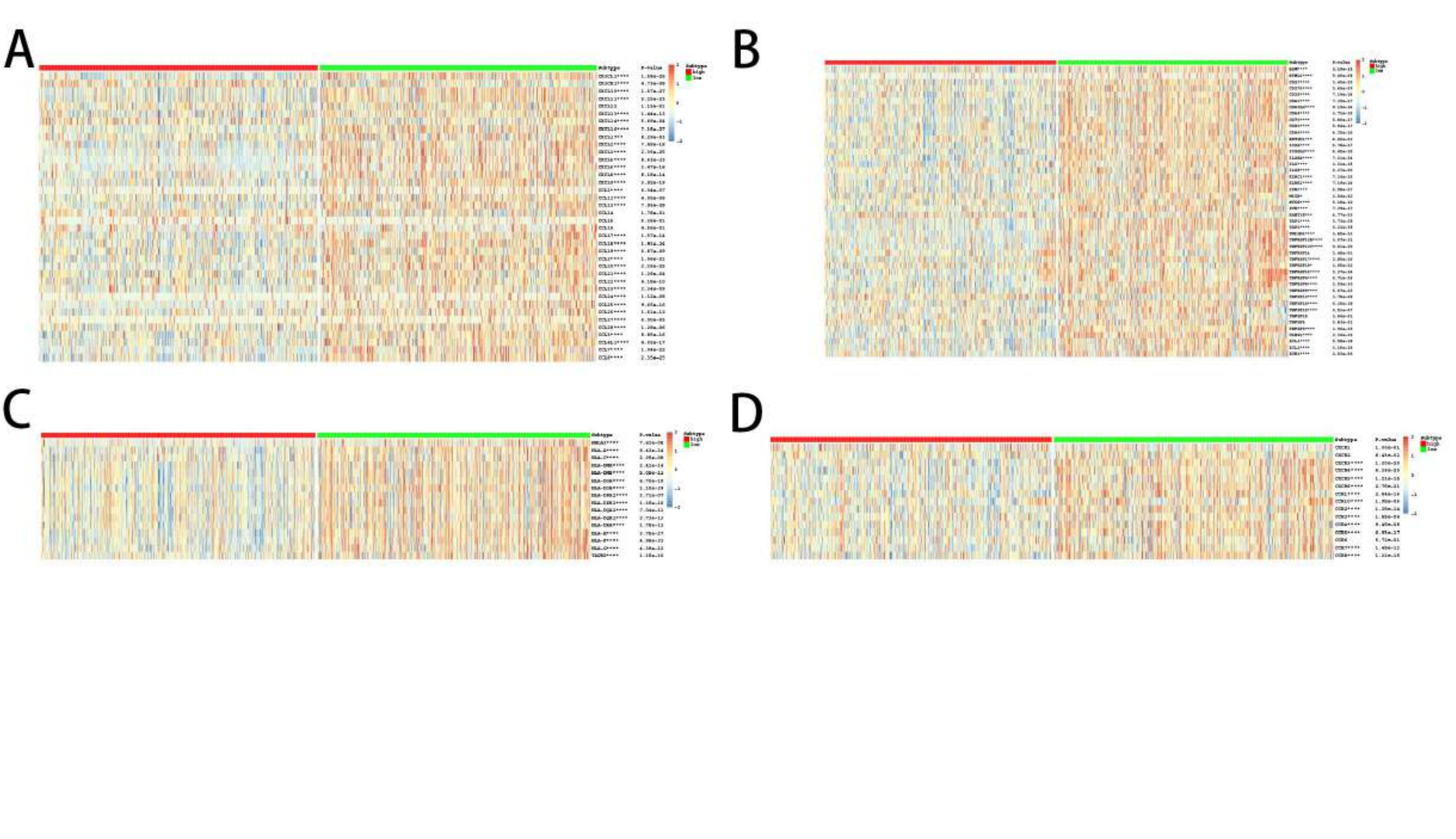

Supplement Figure 3: Correlation between XBP1 and immunomodulators based on TCGA dataset. (A) chemokines; (B) immunostimulators; (C) MHC; (D) receptors.

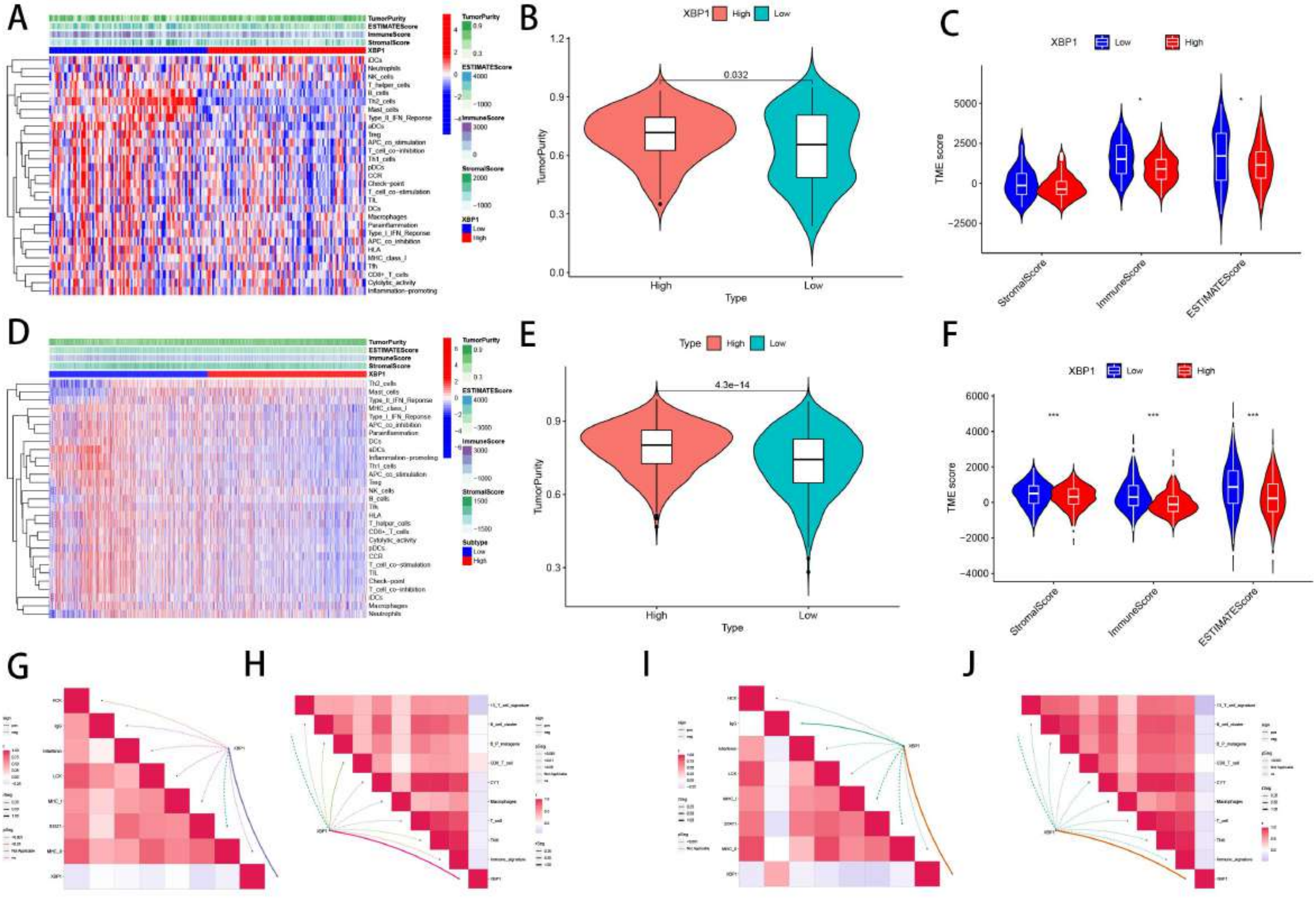
A
B
C
D
E
F
G
H
I
J
TumorPurity
ESTIMATEScore
ImmuneScore
StromalScore
XBP1
Low
High
Subtype
XBP1 High Low
Type High Low
0.032
4.3e−14
TumorPurity
Type
XBP1 Low High
TME score
StromalScore
ImmuneScore
ESTIMATEScore
iDCs
Neutrophils
NK_cells
T_helper_cells
B_cells
Th2_cells
Mast_cells
Type_II_IFN_Reponse
aDCs
Treg
APC_co_stimulation
T_cell_co-inhibition
Th1_cells
pDCs
CCR
Check-point
T_cell_co-stimulation
TIL
DCs
Macrophages
Parainflammation
Type_I_IFN_Reponse
APC_co_inhibition
HLA
MHC_class_I
Tfh
CD8+_T_cells
Cytolytic_activity
Inflammation-promoting

Supplement Figure 4: XBP1 shapes a non-inflamed TME in breast cancer based on GSE25056 and TGCA dataset. Scores of the 29 immune-related gene sets between high and low XBP1 group based on (A) GSE25055 and (D) TGCA dataset; Difference in TumorPurity between high and low XBP1 group based on (B) GSE25055 and (E) TGCA dataset; Difference in TME score between high and low XBP1 group based on (C) GSE25055 and (F) TGCA dataset; Correlations between XBP1 and enrichment scores of inflammatory activation functions based on (G) GSE25055 and (I) TGCA dataset; Correlations between XBP1 and enrichment scores of immunocytes based on (H) GSE25055 and (J) TGCA dataset.

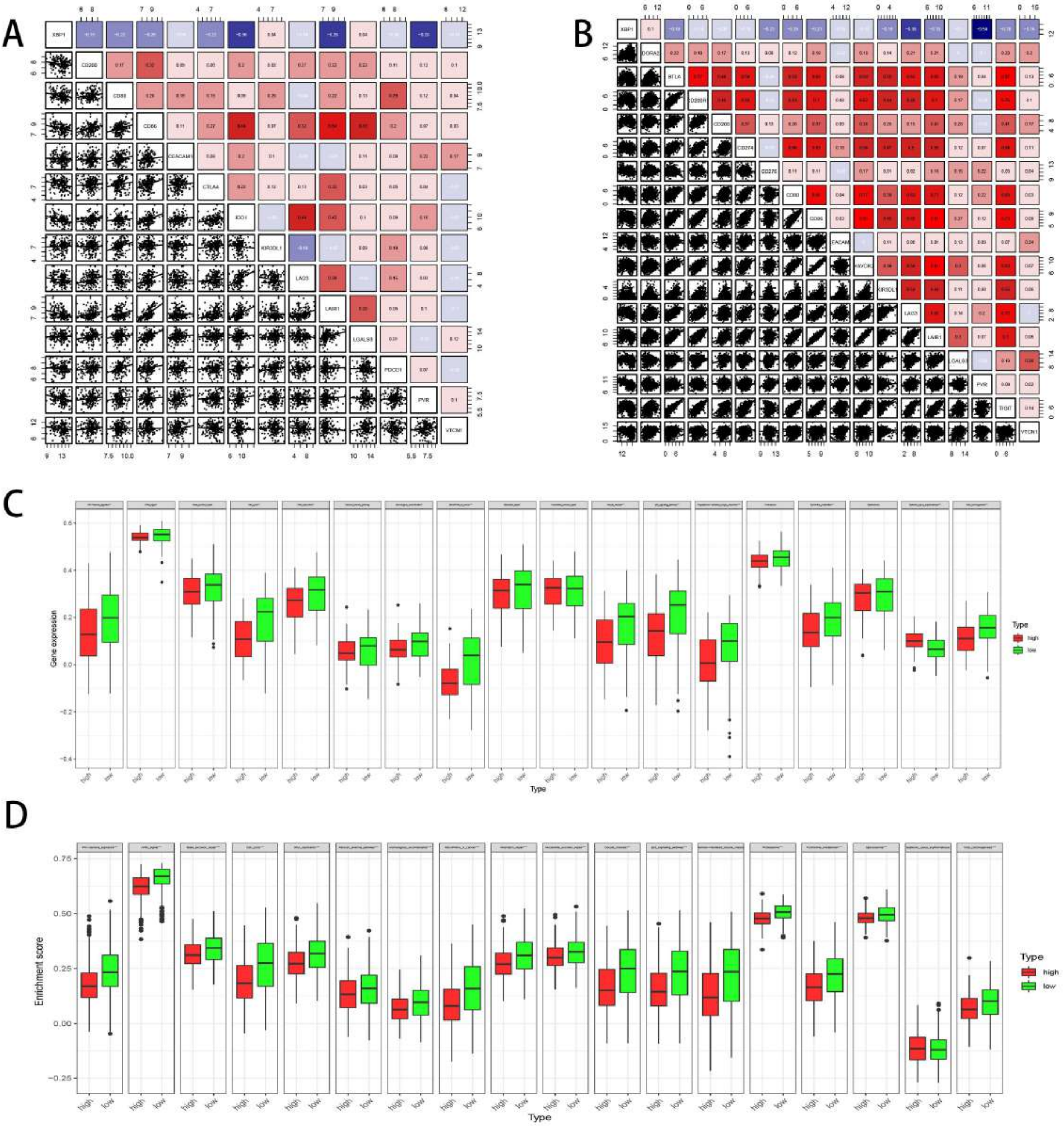
A
XBP1
CD200
CD80
CD86
CEACAM1
CTLA4
IDO1
KIR3DL1
LAG3
LAIR1
LGALS9
PDCD1
PVR
VTCN1
B
XBP1
BTLA
CD200R
CD200
CD274
CD276
CD80
CD86
HAVCR2
LAG3
LAIR1
PVR
TIGIT
VTCN1
C
Gene expression
Type
high
low
D
Enrichment score
Type
high
low

Supplement Figure 5: Correlation between XBP1 and inhibitory immune checkpoints based on GSE25055 and TGCA dataset. Correlation between XBP1 and inhibitory immune checkpoints based on (A) GSE25055 and (B) TGCA dataset; Differences in the enrichment scores of immunotherapy-predicted pathways between high and low XBP1 groups based on (C) GSE25055 and (D) TGCA dataset.

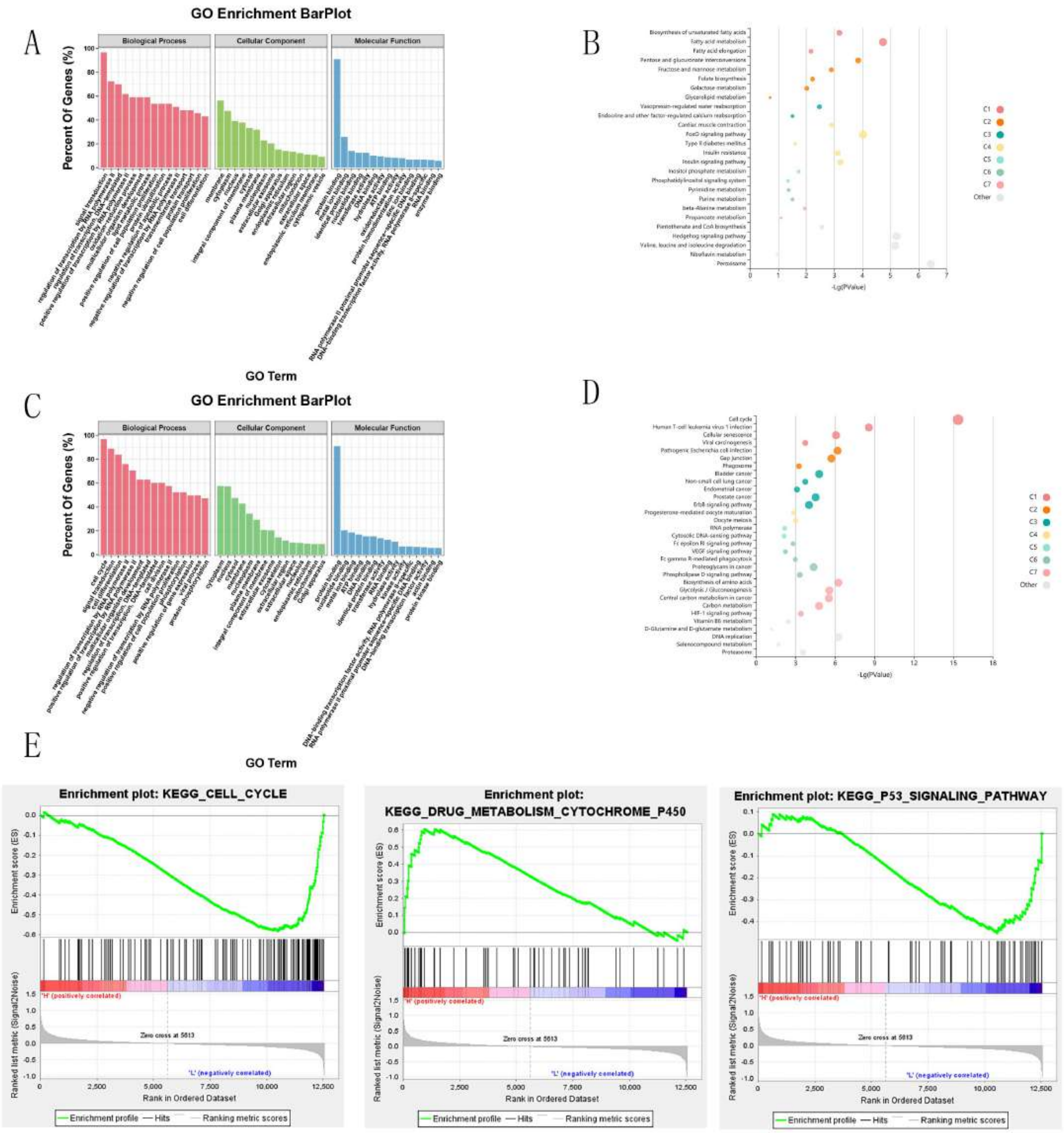
A
GO Enrichment BarPlot
Biological Process
Cellular Component
Molecular Function
Percent Of Genes (%)
GO Term
B
-Lg(PValue)
C1
C2
C3
C4
C5
C6
C7
Other
C
GO Enrichment BarPlot
Biological Process
Cellular Component
Molecular Function
Percent Of Genes (%)
GO Term
D
-Lg(PValue)
C1
C2
C3
C4
C5
C6
C7
Other
E
Enrichment plot: KEGG_CELL_CYCLE
Enrichment plot: KEGG_DRUG_METABOLISM_CYTOCHROME_P450
Enrichment plot: KEGG_P53_SIGNALING_PATHWAY
Enrichment score (ES)
Ranked list metric (Signal2Noise)
'H' (positively correlated)
'L' (negatively correlated)
Zero cross at 5613
Rank in Ordered Dataset
Enrichment profile
Hits
Ranking metric scores

Supplement Figure 6: Functional enrichment analysis based on breast cancer patients received neoadjuvant chemotherapy. (A) Go enrichment of positively correlated significant genes of XBP1 using OmicStudio tool; (B) KEGG enrichment of positively correlated significant genes of XBP1 using KOBAS tool; (C) Go enrichment of negatively correlated significant genes of XBP1; (D) KEGG enrichment of negatively correlated significant genes of XBP1; (E) Gene set enrichment analysis revealed KEGG signaling pathways.
